# Evaluating the association between dietary salt intake and the risk of atrial fibrillation using Mendelian randomization

**DOI:** 10.3389/fnut.2023.1073626

**Published:** 2023-04-06

**Authors:** Sicen Wang, Ye Cheng, Qi Zheng, Xin Su, Yingjian Deng

**Affiliations:** ^1^School of Pharmacy, Health Science Center, Xi’an Jiaotong University, Xi’an, China; ^2^Department of Cardiology, Xiamen Cardiovascular Hospital of Xiamen University, School of Medicine, Xiamen University, Xiamen, China; ^3^Department of Psychology, Xiamen Xianyue Hospital, Xiamen, China

**Keywords:** dietary salt intake, atrial fibrillation, Mendelian randomization, causal association, genetic instrument

## Abstract

**Background:**

Previous studies have suggested that dietary salt intake affects atrial fibrillation (AF); however, the causal association between them still remains unclear. Thus, we conducted this Mendelian randomization (MR) study to explore the correlation between them.

**Methods:**

Genetic instruments for dietary salt intake were from a genome-wide association study (GWAS), which included 462,630 European individuals. Summary-level data for AF were obtained from another published GWAS (22,068 cases and 116,926 controls). The inverse-variance weighting (IVW) method was performed as the primary MR analysis. Multiple MR methods, including Robust Adjusted Profile Score (MR-RAPS), maximum likelihood estimation, and Mendelian randomization pleiotropy residual sum and outlier test (MR-PRESSO) were conducted as complementary analyses. The MR-Egger regression intercept and MR-PRESSO global test were conducted to test potential horizontal pleiotropy. The IVW (Q) method and MR-Egger were performed to detect heterogeneity.

**Results:**

Our results suggested that high dietary salt intake was significantly correlated with increased risk of AF [IVW: odds ratio (OR), 1.36; 95% confidence interval (CI), 1.04–1.77; *p* = 2.25E-02]. The maximum likelihood estimation (OR, 1.37; 95% CI, 1.05–1.78; *p* = 2.09E-02), MR-RAPS (OR, 1.37; 95% CI, 1.03–1.81; *p* = 2.79E-02), and MR-PRESSO method (OR, 1.36; 95% CI, 1.05–1.76; *p* = 2.37E-02) also showed that dietary salt intake was significantly correlated with the risk of AF.

**Conclusion:**

The findings of this study provide robust evidence supporting the correlation between dietary salt intake and the risk of AF. Future studies are required to further clarify this relationship and translate the findings into clinical and public health practice.

## 1. Introduction

Atrial fibrillation (AF) is the most prevalent cardiac arrhythmia worldwide, associated with substantial morbidity and economic burden (1). It is expected that the number of individuals suffering from AF will reach 14 million by the year 2060 in Europe and 16 million in the United States by 2050 (2). The pathogenesis of AF, involving genetic susceptibility and a variety of environmental factors, has become increasingly appreciated. A better understanding of risk factors is critical for the prevention and treatment of AF.

In western diet, adding salt to foods at the table is a common eating habit, which is related to individual salt preference and daily salt intake (3, 4). High salt intake is a well-recognized and modifiable risk factor for cardiovascular diseases (5–7). The World Health Organization recommended that daily salt intake should be less than 5 g to reduce the risk of cardiovascular diseases (8). In the Global Burden of Diseases Study (GBD 2019), high salt diet is one of the major risk factors of AF-related death (9). Studies in recent years suggested that high salt diet may also be a potentially modifiable risk factor for AF. For example, a recent large-scale prospective observational study reported that higher salt intake was related to an increased risk of AF (10), and similar findings have also been demonstrated in another observational study (11). However, the correlation between dietary salt intake and the risk of AF is still unclear and its epidemiology is based on relatively few studies (12). All the available studies exploring the association between dietary salt intake and the risk of AF have been observational, making the analyzes susceptible to potential confounding and reverse causation bias; thus, they are still insufficient to establish the potential causality.

Mendelian randomization (MR) is an epidemiological approach using genetic variants as instrumental variables to assess whether exposure is causally related to the outcome (13). As the genetic variants are randomly assigned and established at the time of conception, MR is less prone to potential confounding and reverse causation bias and has been used extensively to assess causality (14). Therefore, in this present study, we used MR to verify whether high dietary salt intake is related to an increased risk of AF based on summary data from the latest available genome-wide association studies (GWASs).

## 2. Methods

### 2.1. Study design

**Figure 1:**
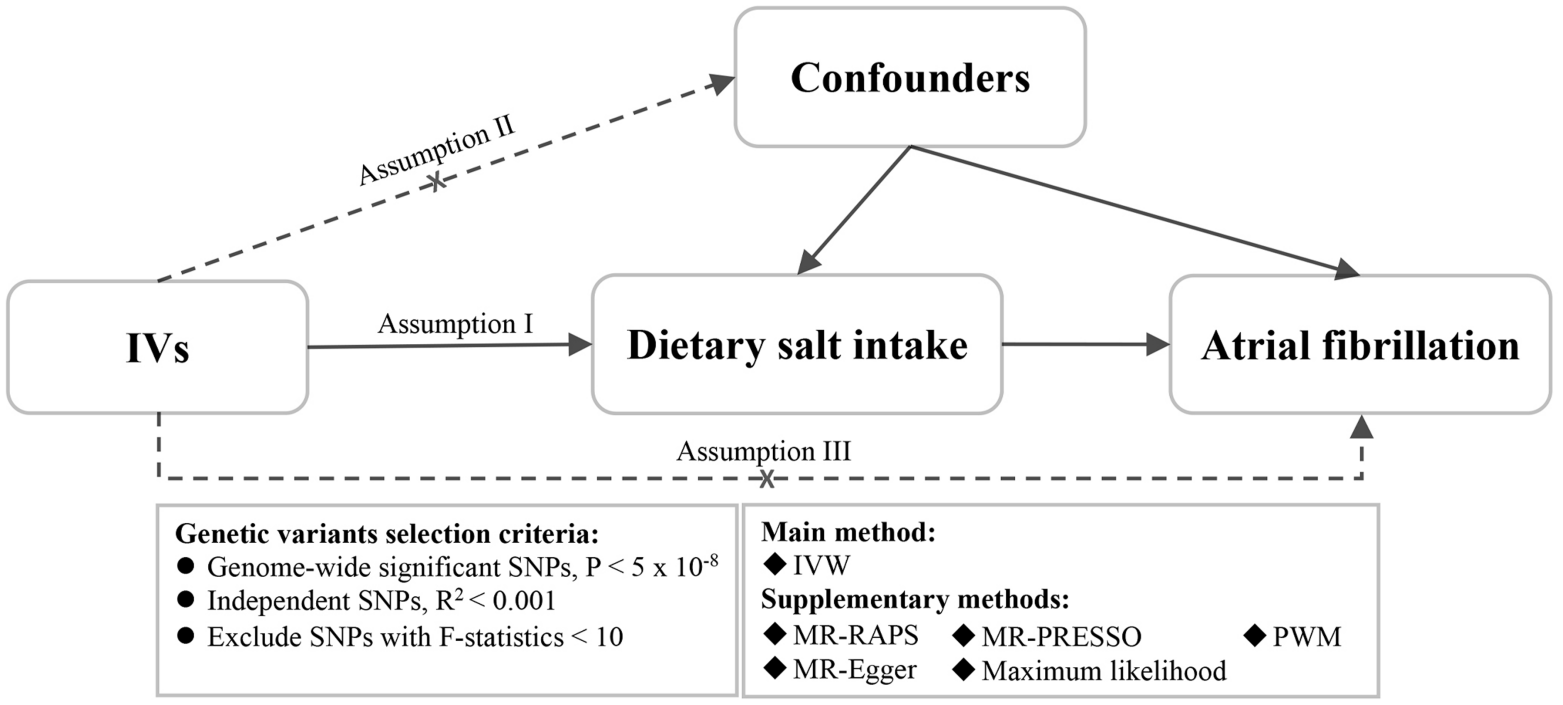
Assumptions of the Mendelian randomization analysis for dietary salt intake and the risk of atrial fibrillation. IVs, instrumental variables; SNPs: single nucleotide polymorphisms; IVW, inverse variance weighted; MR-RAPS, Mendelian randomization robust adjusted profile score; MR-PRESSO: Mendelian randomization pleiotropy residual sum and outlier; PWM, penalized weighted median; MR-Egger, Mendelian randomization-Egger.

The present study was conducted using a two-sample MR design based on summary-level data from independent nonoverlapping populations for dietary salt intake (n = 462,630) and AF (n = 138,994). The validity of our MR study relies on the following three assumptions (Figure 1): (I) genetic variants must be significantly correlated with dietary salt intake (*p* < 5 × 10^−8^); (II) genetic variants must not be correlated with confounding factors; and (III) genetic variants must be correlated with AF only *via* dietary salt intake (15). The original GWASs were approved by the relevant institutional review boards and all the participants provided informed consent.

### 2.2. Data sources

The genetic variants for dietary salt intake were extracted from the latest and largest GWAS (Dataset ID: ukb-b-8,121), including 462,630 individuals in the UK Biobank. UK Biobank is a large-scale populational biomedical database and research resource, which involved about 500,000 participants (aged 40–69 years) (16). All the dietary data were evaluated as categorical variables through questionnaires. The questionnaire included a question: “Do you add salt to your foods? (Do not include salt used in cooking),” and answers were selected from the following five options: never/rarely; sometimes; usually; always; prefer not to answer (4).

Summary-level data for AF were generated from a recent GWAS (Dataset ID: finn-b-I9_AF), which included 138,994 individuals of European descent. The FinnGen study is a global research project aiming at collecting and analyzing the genome and health data of half a million Finns, and has already recruited 224,737 participants (17). The health data was collected from different national health registers, and AF was identified according to the International Classification of Disease-10 (ICD-10) criteria (17).

The details of the datasets included in the present MR study are summarized in Table 1. All the GWAS summary data used in the analyzes of the present MR study were retrieved from the IEU OpenGWAS project^1^.

**Table 1:** Details of studies included in Mendelian randomization analyzes.

Exposure/outcome	GWAS ID	Consortium	Sample size	SNPs number	Ethnicity	Year
Dietary salt intake	ukb-b-8,121	UKB	462,630	9,851,867	European	2018
Atrial fibrillation	finn-b-I9_AF	FinnGen	138,994	16,379,794	European	2021

UKB: United Kingdom Biobank.

### 2.3. Selection of genetic instruments

In the present study, we first selected 106 independent significant single nucleotide polymorphisms (SNPs) that are independent of each other (*R*^2^ < 0.001) and have a genome-wide significant *p* value (*p* < 5 × 10^−8^) for dietary salt intake. With the use of the PhenoScanner database V2 (18, 19), 19 SNPs were identified and removed due to their association with confounders (i.e., hypertension, blood pressure, coronary heart disease, diabetes, and hyperthyroidism) and AF. We further removed 4 SNPs for palindromic and incompatible alleles (rs55897719, rs13084934, rs6443950, and rs976179). Furthermore, the strength of each SNP was measured by F-statistics (*F* = *R*^2^/(1-*R*^2^) × [(N-K-1)/K], where *R*^2^ was the proportion of the exposure explained by the genetic variants, K was the number of included SNPs, and N was the sample size) to avoid weak-instrument bias (*F* > 10 suggested a low probability for weak instrument bias) (20). Eventually, 83 SNPs were selected as instrumental variables in the analyzes; and the F-statistics of these SNPs were all above the threshold of 10 (range from 29.8 to 224.9; Supplementary Table S1).

### 2.4. Statistical analysis

The inverse-variance weighting (IVW) method was performed as the principal MR analytic approach in this study (21). We also conducted several complementary analyzes including the MR-Egger (22, 23), maximum likelihood (21), Robust Adjusted Profile Score (MR-RAPS) (24), penalized weighted median (PWM) (25), and Mendelian randomization pleiotropy residual sum and outlier test (MR-PRESSO) (26) method in the following sensitivity analyzes. The MR-Egger regression intercept and MR-PRESSO global test were performed to examine potential horizontal pleiotropy. We also conducted the IVW (Q) method and MR-Egger to assess the heterogeneity of the data. With regard to the IVW method, a random-effects model would be selected when heterogeneity existed, and a fixed-effects model would be selected when heterogeneity was not significant. In addition, we used MR-PRESSO to identify and, if necessary, correct the possible horizontal pleiotropic outliers in our MR analysis (26). A leave-one-out analysis was carried out to assess the influence of individual variants on the overall results. Statistical analyzes were conducted using R software (version 4.1.2) with the TwoSampleMR (version 0.5.6) (27), mr.raps (28), and MR-PRESSO (26) packages.

## 3. Results

**Figure 2:**
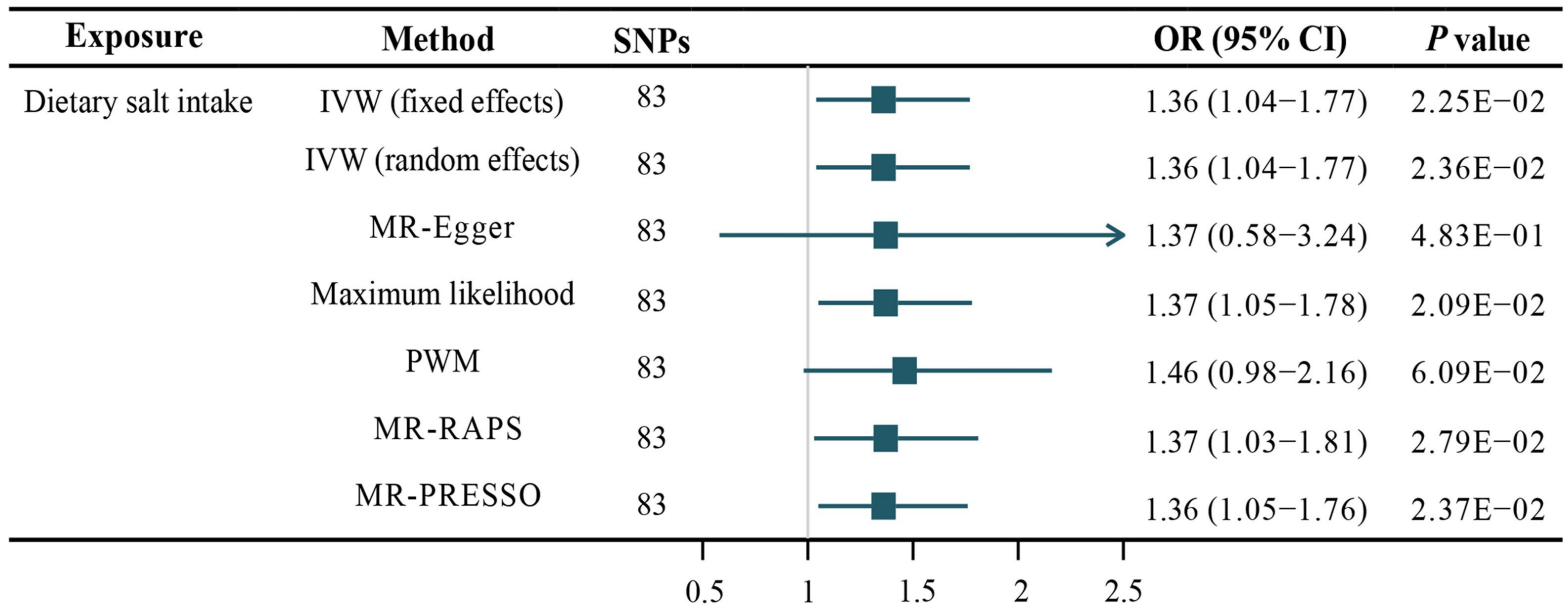
Association of dietary salt intake and the risk of atrial fibrillation. SNPs, single nucleotide polymorphisms; IVW, inverse variance weighted; MR-Egger, Mendelian randomization-Egger; PWM, penalized weighted median; MR-RAPS, Mendelian randomization robust adjusted profile score; MR-PRESSO: Mendelian randomization pleiotropy residual sum and outlier.

**Figure 3:**
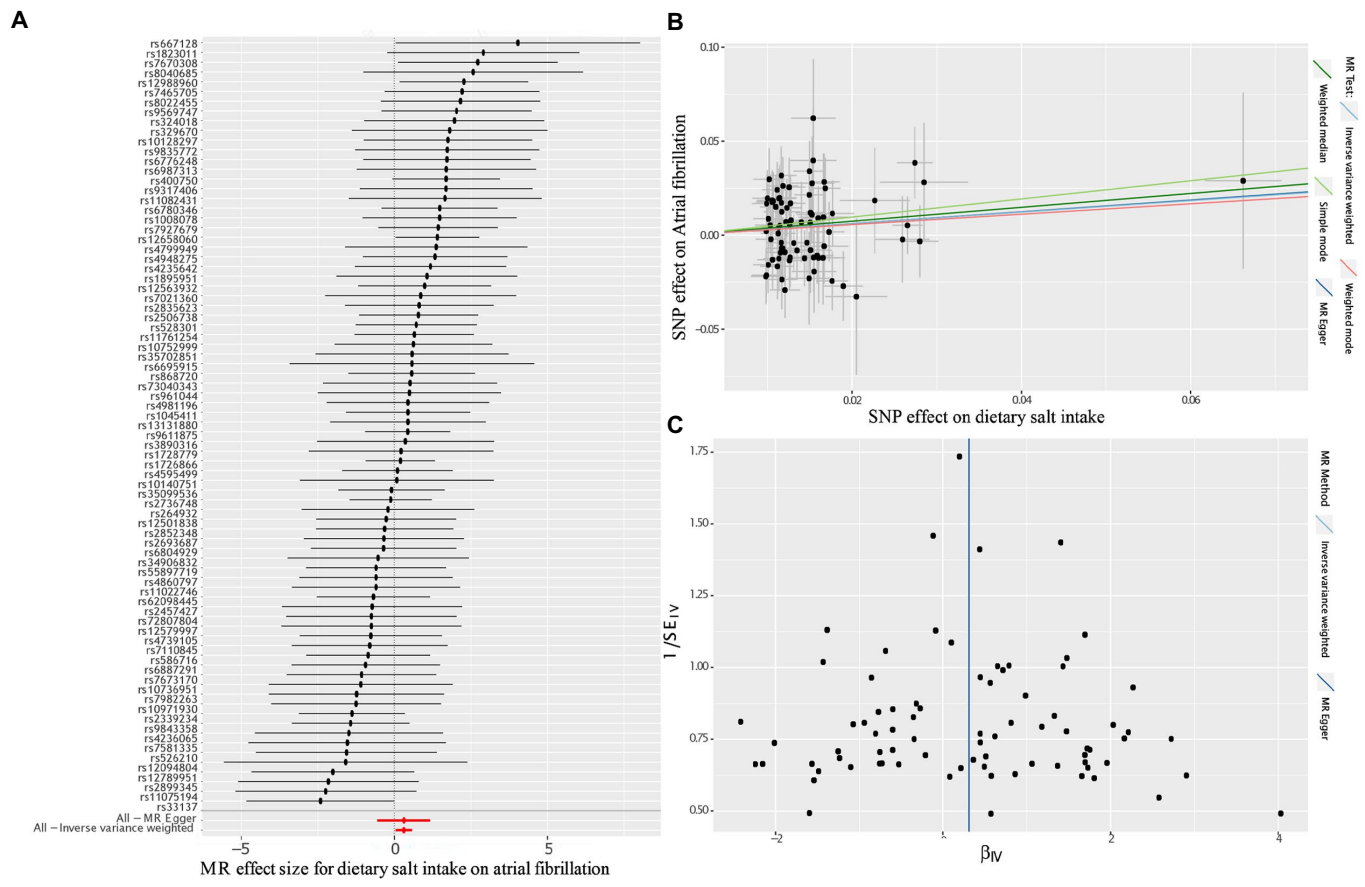
Forest plot, Scatter plot and funnel plot of the effect of dietary salt intake on atrial fibrillation. **(A)** Forest plot shows the odds ratio (OR) and 95% confidence interval (CI) of each SNP assessed in the present study; **(B)** Scatter plot for MR analyzes of the association between dietary salt intake an atrial fibrillation. The slope of each line corresponds to the estimated association of different MR methods; **(C)** Funnel plot shows the estimates of precision (1/SE) and Wald ratios for each SNP; SNP, single nucleotide polymorphism; MR, Mendelian randomization.

The fixed-effects IVW method was used as our principal MR analytic approach. The fixed-effects IVW method suggested that genetically determined dietary salt intake was significantly correlated with the risk of AF [odds ratio (OR),1.36; 95% confidence interval (CI), 1.04–1.77; *p* = 2.25E-02; Figure 2]. Similarly, the random-effects IVW (OR, 1.36; 95% CI, 1.04–1.77; *p* = 2.36E-02), maximum likelihood estimation (OR, 1.37; 95% CI, 1.05–1.78; *p* = 2.09E-02), MR-RAPS (OR, 1.37; 95% CI, 1.03–1.81; *p* = 2.79E-02), and MR-PRESSO method (OR, 1.36; 95% CI, 1.05–1.76; *p* = 2.37E-02) also showed that high dietary salt intake was significantly correlated with an increased risk of AF (Figure 2). A forest plot of each dietary salt intake SNP associated with the risk of AF is demonstrated in Figure 3A. No outlier SNPs were detected in the MR-PRESSO analysis. The estimated effect of each SNP on both the exposure (dietary salt intake) and AF were determined using different MR analysis methods (Figure 3B). The funnel plot suggested that no SNPs exhibited horizontal pleiotropy (Figure 3C).

The Cochran’s Q test and MR-Egger method found no significant heterogeneity for these 83 SNPs (Cochran’s Q test: *p* = 0.44, MR-Egger: *p* = 0.41; Table 2). Furthermore, the MR-PRESSO global test and MR-Egger regression intercept revealed the absence of horizontal pleiotropy (MR-PRESSO global test: *p* = 0.47; MR-Egger regression intercept: *p* = 0.99; Table 2). The leave-one-out analysis showed that rs12658060 might have a potential impact on our IVW results (Supplementary Figure S1).

**Table 2:** Heterogeneity and pleiotropy estimates for the associations between dietary salt intake and AF.

Exposures	Heterogeneity test (IVW)		Heterogeneity test (MR-Egger)		MR-PRESSO global test	MR Egger intercept test		
	*Q*	*p*-value	Q	*P*-value	*P-*value	I	SE	*P*-value
Dietary salt intake	79.19	0.44	79.19	0.41	0.47	<0.01	0.01	0.99

IVW, inverse variance weighted; MR-Egger, Mendelian Randomization-Egger; AF. Atrial fibrillation.

## 4. Discussion

The present MR study was conducted to explore the correlation between dietary salt intake and the risk of AF. Our findings demonstrated that high dietary salt intake is significantly correlated with an increased risk of AF (IVW: OR, 1.36; *p* = 2.25E-02). F-statistics of each SNP were above the threshold of 10, suggesting that the selected SNPs were robust instruments for dietary salt intake. Concordant results from multiple MR methods increased the robustness of our findings.

The correlation between salt intake and the risk of AF is inconsistent in previous observational studies. A prospective study on Finland’s population with a follow-up period of 19 years indicated that high dietary salt intake might increase the future risk of AF [hazard ratio (HR) per SD increase, 1.3–1.4] (11). Another recent observational and prospective study on the relationship between daily salt intake and AF risk, which involved 257,545 females and 215,535 males, suggested a U-shaped association between dietary salt consumption and the risk of AF in males and a tendency for a J-shaped association in females (10). The results indicated that increased dietary salt intake above a certain physiological level was associated with an increased risk of AF. Similarly, the findings from the present MR study also demonstrated that high dietary salt intake increases the risk of AF. However, a large cross-sectional study suggested that salt intake was not correlated with the risk of AF after an adjusted multivariate analysis by Cox proportional hazard regression analysis (29). Moreover, one of the latest meta-analyzes, which involved over 1.4 million individuals, showed that high salt intake did not significantly increase the risk of AF (RR, 1.02; 95% CI, 0.96–1.07) (12). But it is noteworthy that this meta-analysis had considerable heterogeneity (*P* for heterogeneity = 0.074; *I*^2^ = 53.1%) (12), which might explain some of the discrepancies in the results. However, studies were almost from developed western countries, and studies from developing countries are still lacking.

The underlying mechanisms of the correlation between salt intake and AF, however, are still elusive. Previous findings have shown that high salt intake is correlated with hypertension and cardiovascular disease risk, which are important risk factors for AF (30–32). Sodium is the most important extracellular cation; excessive salt may change the stretch of cardiac tissue, triggering AF episodes (33–35). Hirota et al. reported that the reduction of salt intake was related to decreased levels of B-type natriuretic peptide, which might be potentially beneficial to AF management (36). Recently, Li et al. found that excess dietary salt intake was related to myocardial remodeling, as well as the impairment of cardiac function and myocardial viability, and inflammation perhaps plays a role in these relationships (37). Previous research also found that high salt intake was correlated with cardiomyocyte hypertrophy, interstitial fibrosis, and cardiac dysfunction (38–40). Lader et al. found that a salt-induced increase in blood pressure could lead to the activation of the K_ATP_ channel and thus increased arrhythmia inducibility (41). A recent study by Harada et al. found that QRS and QT intervals were prolonged and AERP shortened in Dahl rats fed a high-salt diet (38). Meanwhile, a growing body of evidence suggests the correlation between salt intake and the rennin-angiotensin-aldosterone system, which plays a critical role in the development of AF (42–44).

In this study, we further demonstrated that high dietary salt intake is correlated with increased AF risk. Our results are potentially more reliable and robust as we used the statistics from large-scale GWASs, with no obvious horizontal pleiotropy, heterogeneity, or outliers observed. Furthermore, the MR design could reduce the impact of confounders and reverse causality. However, there were still some limitations to this work. First, the recall bias and measurement error in self-reported dietary salt intake are unavoidable. Second, we assumed a linear correlation between dietary salt intake and the risk of AF, which may be more complex in reality (10). Third, only summary-level data were used in our study, which precluded us from further stratifying patients into different subgroups. Fourth, the self-reported frequency of adding salt to foods might not provide accurate quantitative information on daily intake of salt. In addition, the GWASs data used in the present study were restricted to participants of European descent, which could limit the study’s generalizability to other populations.

## 5. Conclusion

Overall, the present MR study demonstrated that genetically determined high dietary salt intake was significantly correlated with an increased risk of AF. Future studies will be needed to further clarify this relationship and confirm the generalizability of our results to more socioeconomically and ethnically diverse populations.

## Data availability statement

The original contributions presented in the study are included in the article/Supplementary material, further inquiries can be directed to the corresponding author.

## Ethics Statement

Ethical approval was not provided for this study on human participants because the original GWASs were approved by the relevant institutional review boards and all the participants provided informed consent. The patients/participants provided their written informed consent to participate in this study. Written informed consent was obtained from the individual(s) for the publication of any potentially identifiable images or data included in this article.

## Author Contributions

YJD, QZ, and YC designed and conceptualized the study. YJD, YC, QZ, and XS searched databases and analyzed the data. SCW drafted the original draft of the manuscript. YJD and XS reviewed and edited the manuscript. All authors contributed to the article and approved the submitted version.

## Conflict of interest

The authors declare that the research was conducted in the absence of any commercial or financial relationships that could be construed as a potential conflict of interest.

## Publisher’s note

All claims expressed in this article are solely those of the authors and do not necessarily represent those of their affiliated organizations, or those of the publisher, the editors and the reviewers. Any product that may be evaluated in this article, or claim that may be made by its manufacturer, is not guaranteed or endorsed by the publisher.

## Footnotes

^1^https://gwas.mrcieu.ac.uk/
