# Supplementary figures and images for: Evaluating the association between dietary salt intake and the risk of atrial fibrillation using Mendelian randomization
Source: Front Nutr. 2023 Apr 6;10:1073626. doi: 10.3389/fnut.2023.1073626 (PMC10117818; doi:10.3389/fnut.2023.1073626)

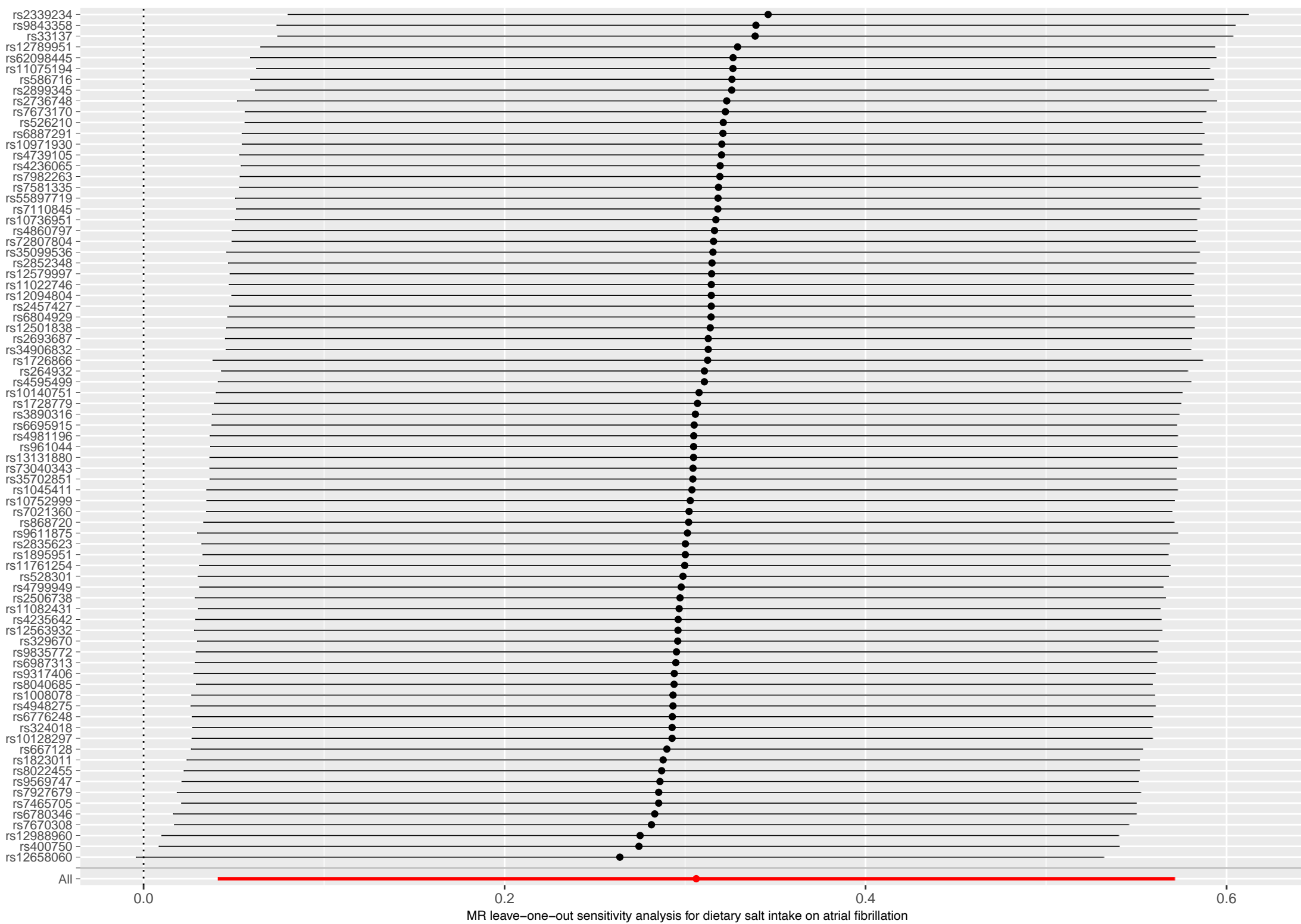

Supplement: Supplementary file 1 [file Data_Sheet_1.PDF]
